# Gene expression of key regulators of mitochondrial biogenesis is sex dependent in mice with growth hormone receptor deletion in liver

**DOI:** 10.18632/aging.100733

**Published:** 2015-03-30

**Authors:** Ilona Zawada, Michal M. Masternak, Edward O. List, Michael B. Stout, Darlene E. Berryman, Andrzej Lewinski, John J. Kopchick, Andrzej Bartke, Malgorzata Karbownik-Lewinska, Adam Gesing

**Affiliations:** ^1^ Department of Oncological Endocrinology, Medical University of Lodz, 90-752 Lodz, Poland; ^2^ College of Medicine, Burnett School of Biomedical Sciences, University of Central Florida, Orlando, FL 32827, USA; ^3^ Department of Head and Neck Surgery, The Greater Poland Cancer Centre, 61-866 Poznan, Poland; ^4^ Edison Biotechnology Institute, Ohio University, Athens, OH 45701, USA; ^5^ Department of Specialty Medicine, Ohio University, Athens, OH 45701, USA; ^6^ Robert and Arlene Kogod Center on Aging, Mayo Clinic, Rochester, MN 55905, USA; ^7^ School of Applied Health Sciences and Wellness, College of Health Sciences and Professions, Ohio University, Athens, OH 45701, USA; ^8^ Department of Biomedical Sciences, Heritage College of Osteopathic Medicine, Ohio University, Athens, OH 45701, USA; ^9^ Department of Endocrinology and Metabolic Diseases, Medical University of Lodz, 93-338 Lodz, Poland; ^10^ Department of Endocrinology and Metabolic Diseases, Polish Mother's Memorial Hospital – Research Institute, 93-338 Lodz, Poland; ^11^ Department of Internal Medicine, Geriatrics Research, Southern Illinois University School of Medicine, Springfield, IL 62794, USA

**Keywords:** mitochondrial biogenesis, gene disruption, growth hormone receptor, knockout mice, tissue-specific gene disruption, sexual dimorphism

## Abstract

Mitochondrial biogenesis is an essential process for cell viability. Mice with disruption of the growth hormone receptor (GHR) gene (*Ghr* gene) in the liver (LiGHRKO), in contrast to long-lived mice with global deletion of the *Ghr* gene (GHRKO), are characterized by lack of improved insulin sensitivity and severe hepatic steatosis. Tissue-specific disruption of the GHR in liver results in a mouse model with dramatically altered GH/IGF1 axis. We have previously shown increased levels of key regulators of mitochondrial biogenesis in insulin-sensitive GHRKO mice. The aim of the present study is to assess, using real-time PCR, the gene expression of key regulators of mitochondrial biogenesis (*Pgc1α, Ampk, Sirt1, Nrf2* and *Mfn2*) and a marker of mitochondrial activity (*CoxIV*) in brains, kidneys and livers of male and female LiGHRKO and wild-type (WT) mice. There were significant differences between males and females. In the brain, expression of *Pgc1α, Ampk, Sirt1, Nrf2* and *Mfn2* was lower in pooled females compared to pooled males. In the kidneys, expression of *Ampk* and *Sirt1* was also lower in female mice. In the liver, no differences between males and females were observed. Sexual dimorphism may play an important role in regulating the biogenesis of mitochondria.

## INTRODUCTION

The biogenesis of mitochondria is a process by which new mitochondria are formed and is critical for cell viability [1]. Mitochondria are complex eukaryotic organelles that play a crucial role in energy homeostasis and metabolism. Disruption of mitochondrial biogenesis may lead, *via* impaired oxidative stress resistance and maintenance of energy production, to the development of numerous degenerative and metabolic diseases [2, 3]. On the contrary, increased level of mitochondrial biogenesis may prevent aging [4].

There are numerous key regulators of mitochondrial biogenesis, including peroxisome proliferator-activated receptor gamma (PPARγ) co-activator 1 alpha (PGC1α), AMP-activated protein kinase (AMPK), sirtuin 1 (SIRT1), nuclear respiratory factor 2 (NRF2) and mitofusin 2 (MFN2). Additionally, cytochrome c oxidase (COX) is one of the mitochondrial activity markers.

PGC1α is the master regulator of mitochondrial biogenesis. This transcriptional coactivator coordinates the actions of several transcription factors and thereby controls mitochondrial biogenesis (reviewed by [3]). AMPK plays an important role as a cellular energy sensor and is activated by an increase in intracellular adenosine monophosphate (AMP)/adenosine triphospha-te (ATP) ratio [5]. Sirtuin 1, sometimes referred to as a nutrient deprivation sensor, belongs to the sirtuin (NAD^+^–dependent deacetylases) family and stimulates mitochondrial biogenesis *via* PGC1α deacetylation [3]. NRF2 is a nuclear-encoded transcription factor that binds and activates various mitochondrial genes required for electron transport and oxidative phosphorylation [6]. MFN2 plays an essential role in mitochondrial fusion and maintenance of the mitochondrial network architecture [7, 8], which is essential for mitochondrial activity and biogenesis.

Long-lived mice with targeted global disruption of the growth hormone (GH) receptor (GHR) gene (*Ghr* gene) (GHRKO; GHR knockout; Laron dwarfs; *Ghr*−/−) [9] are dwarf, obese and insulin sensitive [10]. We have previously shown increased expression of key regulators of mitochondrial biogenesis, including PGC1α, AMPK, SIRT1, eNOS and MFN2 in the kidneys and heart of GHRKO mice [11-13]. Moreover, GHRKO mice have decreased levels of pro-apoptotic factors [13-16] and decreased thyroid follicle size [17] with mild thyroid hypofunction. Interestingly, preservation of cognitive function in aging GHRKO mice was also observed [10]. Results obtained in these and in GH-deficient mice suggest an essential role of GH-induced intracellular signaling in lifespan regulation [18].

Insulin-like growth factor 1 (IGF1) mediates many of GH's physiological effects. IGF1 is primarily produced in the liver and acts systemically, although other tissues also may produce IGF1 where it may act locally (e.g., [19]). Therefore, suppression of GH signaling selectively in different tissues emerges as a very promising experimental approach to better understand the mechanisms involved in regulating the effects of the GH/IGF1 axis, and presumably in lifespan extension. Thus, mice with tissue-specific ablation of the *Ghr* gene recently have been generated [20-25], including mice with tissue-specific deletion of GHR (and disruption of GH signaling) in the liver (LiGHRKO). LiGHRKO mice have decreased body fat, severely reduced levels of circulating IGF1 and concurrently higher GH plasma levels [20, 25]. It is of particular interest that LiGHRKO mice show an absence of improved insulin sensitivity and severe hepatic steatosis [20, 25], which is in contrast to mice with global deletion of GHR. Both sexes of LiGHRKOs are characterized by elevated fasting blood glucose, while fasting insulin is higher only in males. Moreover, males have normal glucose tolerance and mild insulin resistance, while females are glucose intolerant and insulin resistant [25].

A significant alterations in the weights of different organs (e.g., brain, kidneys and liver) between control and LiGHRKO mice were previously described [20, 25], suggesting potential differences in apoptosis as well as in mitochondrial function regulation, because mitochondria are known to play an important role in regulation of the process of apoptosis. Importantly, our recent studies demonstrated a sex-dependent expression of apoptosis-related genes in the brain and kidneys of LiGHRKO mice without any significant main effects of sex in the liver [26]. Additionally, analysis of expression of the examined genes in the brains is related to the reported preservation of cognitive function in aging GHRKO mice [10]. For all these reasons, we decided to assess the effect of liver-specific *Ghr* gene disruption on gene expression of key regulators of mitochondrial biogenesis (*Pgc1α*, *Ampk*, *Sirt1*, *Nrf2*, *Mfn2*) and a marker of mitochondrial activity (*CoxIV*) in brains, kidneys and livers of males and females 22-month old LiGHRKO mice compared to males and females wild-type animals.

We decided to use old mice for the following reasons. First of all, in our paper published recently [26], we have reported the sex dependence of the expression of apoptosis-related genes in 22-month old LiGHRKO mice. On the contrary, we did not find any differences in apoptotic factors gene expression in younger 9-month-old mice (unpublished data). Thus, knowing the above-mentioned important role of mitochondria in the apoptosis regulation, we decided to analyze the gene expression of the mitochondrial biogenesis-related factors in 22-month old LiGHRKO mice.

## RESULTS

### Gene expression of key regulators of mitochondrial biogenesis in the brain

**Figure 1:**
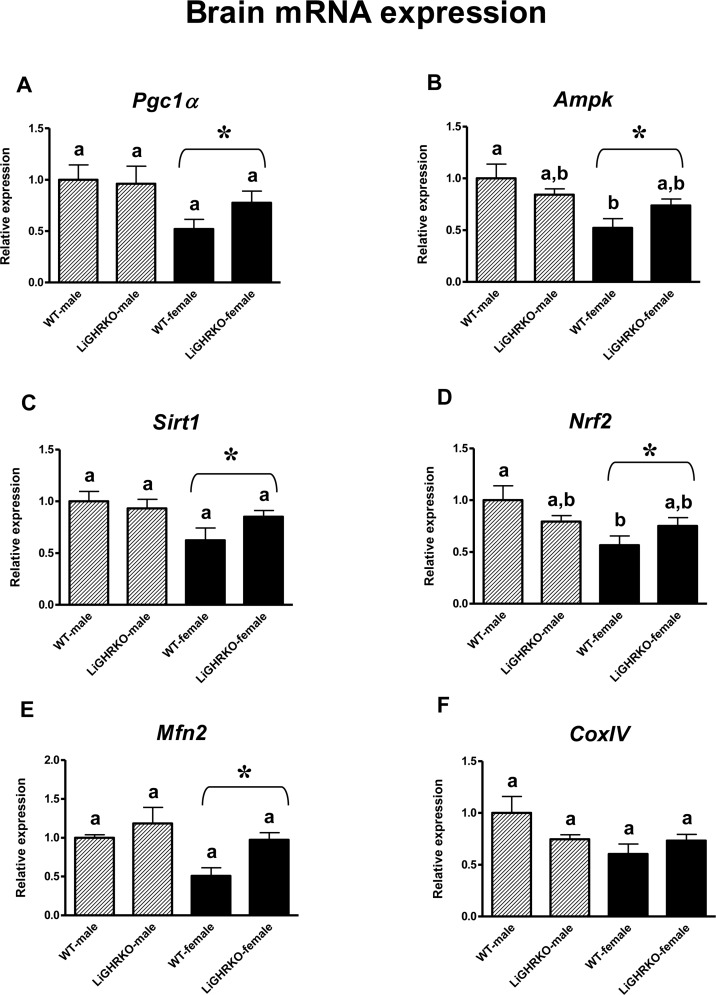
Brain gene expression of key regulators of mitochondrial biogenesis Brain mRNA expression of *Pgc1α* (**A**), *Ampk* (**B**), *Sirt1* (**C**), *Nrf2* (**D**), *Mfn2* (**E**) and *CoxIV* (**F**) in male and female of wild-type (WT) and liver-specific growth hormone receptor knockout (LiGHRKO) mice. Each group consists of 7 animals. The data from real-time PCR were normalized by the housekeeping gene β2-microglobulin (*B2M*) and shown as a relative expression. Values are means ± SEM. a, b – values that do not share the same letter in the superscript are statistically significant (p<0.05). * – p<0.05 vs. male mice (the significance for sex). There are the following significant p values: *Pgc1α*: 0.022, *Ampk*: 0.004, *Sirt1*: 0.021, *Nrf2*: 0.021, *Mfn2*: 0.022.

In the brain, gene expression of *Pgc1α*, *Ampk*, *Sirt1*, *Nrf2* and *Mfn2* was lower in pooled females than in pooled males (p=0.022, p=0.004, p=0.021, p=0.021, p=0.022, respectively) (Figures 1A-E), demonstrating a significant sex effect. *CoxIV* showed a tendency for lower gene expression in female brains (p=0.055) (Figure 1F). Moreover, *Ampk* and *Nrf2* expression was lower in brains of WT-females compared to WT-males (p=0.007 and p=0.024 respectively, with genotype*sex interactions reaching borderline statistical significance – p=0.054 and p=0.053, respectively) (Figures 1B and 1D). There was a weak and not statistically significant tendency for expres-sion levels of all examined genes to be greater in female LiGHRKO mice than in WT-females (Figures 1A-F).

### Gene expression of key regulators of mitochondrial biogenesis in the kidneys

**Figure 2:**
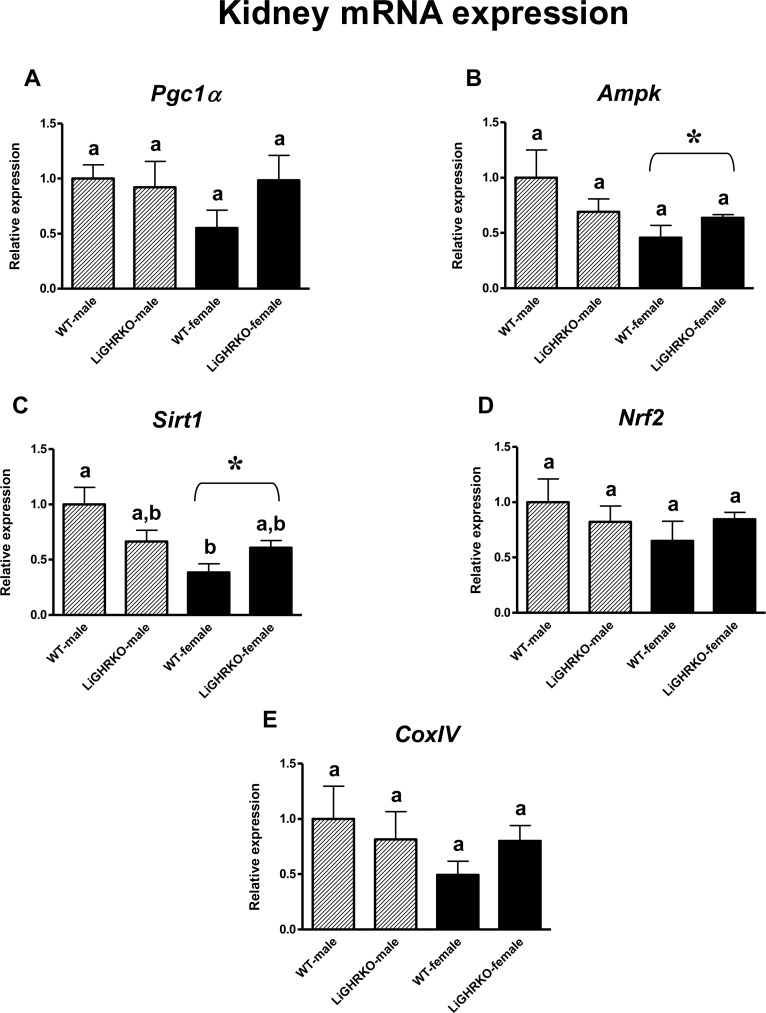
Renal gene expression of key regulators of mitochondrial biogenesis Kidney mRNA expression of *Pgc1α* (**A**), *Ampk* (**B**), *Sirt1* (**C**), *Nrf2* (**D**) and *CoxIV* (**E**) in male and female of wild-type (WT) and liver-specific growth hormone receptor knockout (LiGHRKO) mice. Each group consists of 7 animals. The data from real-time PCR were normalized by the housekeeping gene β2-microglobulin (*B2M*) and shown as a relative expression. Values are means ± SEM. a, b – values that do not share the same letter in the superscript are statistically significant (p<0.05). * – p<0.05 vs. male mice (the significance for sex). There are the following significant p values: *Ampk*: 0.041, *Sirt1*: 0.003.

In the kidneys, a decrease in gene expression of two key regulators of mitochondrial biogenesis (*Ampk* and *Sirt1)* in female compared to male mice was detected (a significant sex effect; p=0.041, p=0.003, respectively) (Figures 2B-C). Moreover, renal *Sirt1* gene expression was lower in WT-females than in WT-males (p=0.003) (Figure 2C). On the contrary, there were no differences in the expression of *Pgc1α*, *Nrf2* and *CoxIV* between male and female kidneys (p=0.366, p=0.315, p=0.242) (Figures 2A, 2D and 2E). Similarly to the results obtained in the brain, there appeared to be a weak tendency for increased gene expression levels of the examined factors in female LiGHRKO mice as compared with WT-females (Figures 2A-E).

### Gene expression of key regulators of mitochondrial biogenesis in the liver

**Figure 3:**
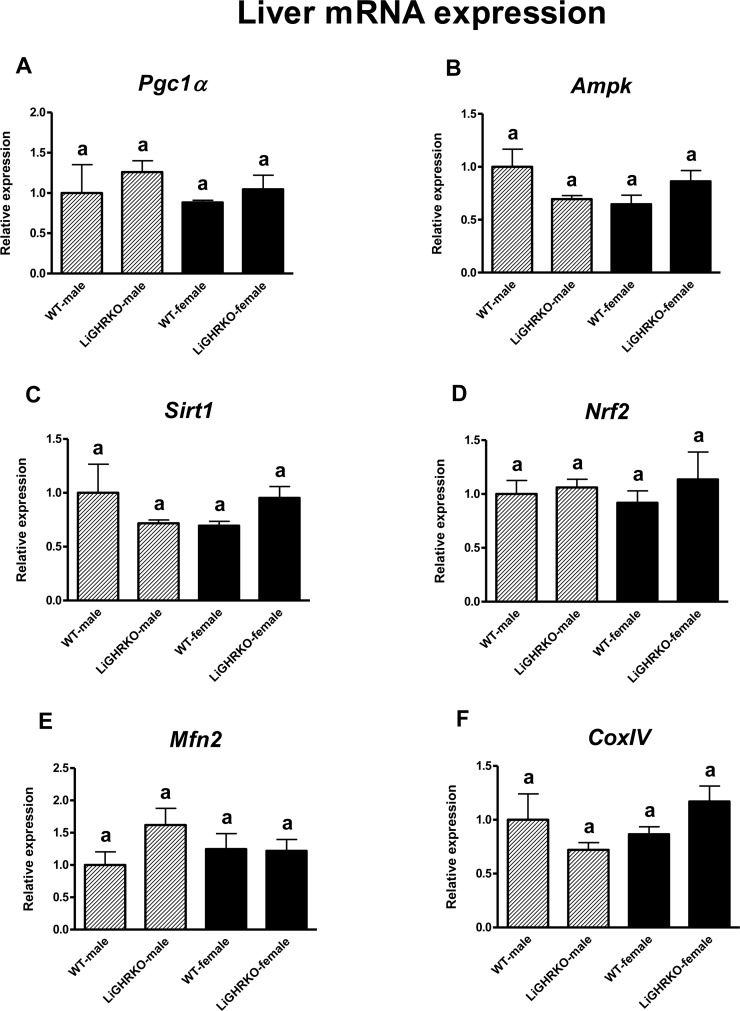
Hepatic gene expression of key regulators of mitochondrial biogenesis Liver mRNA expression of *Pgc1α* (**A**), *Ampk* (**B**), *Sirt1* (**C**), *Nrf2* (**D**), *Mfn2* (**E**) and *CoxIV* (**F**) in male and female of wild-type (WT) and liver-specific growth hormone receptor knockout (LiGHRKO) mice. Each group consists of 7 animals. The data from real-time PCR were normalized by the housekeeping gene β2-microglobulin (*B2M*) and shown as a relative expression. Values are means ± SEM. a – values that share the same letter in the superscript are not statistically significant.

Intriguingly, in the liver, neither sex nor genotype significantly affected mRNA levels of the examined factors (*Pgc1α*, *Ampk*, *Sirt1*, *Nrf2*, *Mfn*2 and *CoxIV*; for sex – p=0.448, p=0.383, p=0.790, p=0.979, p=0.739, p=0.283, respectively and for genotype – p=0.335, p=0.677, p=0.921, p=0.371, p=0.215, p=0.938, respectively) (Figures 3A-F).

## DISCUSSION

Previous studies have shown increased levels of key regulators of mitochondrial biogenesis in mice with global GHR knockout [11, 12]. This increase was interpreted as a potentially beneficial characteristic of long-lived mice with global GHR deletion [13]. Therefore, one could envision that similar changes would be detected in the liver of mice with the same genetic intervention limited to the hepatic tissue (LiGHRKO). Surprisingly, deletion of the GHR only in the liver did not lead to any differences in gene expression of key regulators of mitochondrial biogenesis in this organ compared to wild-type mice. Thus, a main significant genotype effect has not been found. In two organs in which *Ghr* gene was not deleted, namely in brains and kidneys, there were also no significant effects of the genotype. These results may appear counterintuitive in the context of findings in global GHRKO mice, although it is known that LiGHRKO mice, in contrast to GHRKO animals, do not share the same phenotype in many aspects [20, 25], and have no change in lifespan (do not live longer) [27]. Furthermore, one should emphasize that our recent studies on the expression of apoptosis-related genes in LiGHRKO mice have also revealed an absence of genotype effect [26]. Apparently, effects of global GHR deletion differ from the effects of liver-specific GHR knockout. The observed differences strongly emphasize the necessity of further studies on the GH signaling regulation to elucidate which mechanisms involved in this process may play the most crucial role and why the disruption of the GHR in one tissue or organ may have completely opposite effects compared to global GHR deletion.

Besides global GHRKO mice, the levels of key regulators of mitochondrial biogenesis have not yet been assessed in other long-lived mouse strains. However, there are studies on mitochondrial content and biogenesis in human subjects from the Leiden Longevity Study [28]. The authors have shown that the offspring of nonagenarians had lower mitochondrial DNA (mtDNA) content compared to age-matched controls, whereas nonagenarian parents had the lowest content of mtDNA [28]. Thus, mtDNA has been shown to be negatively associated with familial longevity. The content of mtDNA may be considered a marker for the cellular amount of mitochondria and reflects the balance between mitochondrial biogenesis and removal of damaged mitochondria. Results of the study performed by Passtoors et al. [29] have demonstrated that familial longevity is also associated with reduced expression of genes involved in the mTOR pathway. It is known that this signaling pathway positively regulates genes involved in mitochondrial biogenesis, e.g., PGC1α [3]. Therefore, one could hypothesize that unaltered levels of key regulators of mitochondrial biogenesis in LiGHRKO mice, as seen in the present study, do not need to be considered a detrimental feature of these mutant mice. Observations by Malik and Czajka [30] showing opposite (decreased or increased) alterations in mtDNA content in numerous diseases, including those related to age (e.g., cancer or diabetes) also may support this hypothesis. Clearly, further work is required to fully elucidate the role of mitochondrial biogenesis regulation in the lifespan extension.

As noted above, direction of changes in levels of key regulators of mitochondrial biogenesis in LiGHRKO mice differs from those previously demonstrated in GHRKO mutants. Moreover, observations in mice with tissue-specific deletion of the GHR (not only limited to the liver) point to different characteristics of these mutants compared to global GHRKO animals. For example, there is a lack of glucose homeostasis improvement in fat-specific GHRKO (FaGHRKO) mice [24]. Moreover, in contrast to global GHRKOs, mice with GHR deletion in muscles (MuGHRKO) are characterized by insulin resistance and glucose intolerance [21]. GHR disruption in pancreatic beta-cells can lead to impaired insulin secretion [22]. Therefore, one could hypothesize that global GHR disruption with its numerous beneficial effects, but not tissue-specific knockout of *Ghr* gene, may play a crucial role in lifespan extension and resistance to the development of cancer and diabetes seen in GHRKO dwarfs [18].

In the present study, a clear difference between sexes (a main significant sex effect) was demonstrated. Namely, our findings have shown that gene expression of key regulators of mitochondrial biogenesis was lower in females compared to male mice. Importantly, the results of recently published studies seem to support a hypothesis suggesting a potential role of sexual dimorphism in the regulation of biogenesis of mitochondria and may confirm the decreased intensity of this important process in females compared to males. Sex-dependence of mitochondrial biogenesis was observed by Drake et al. [31] in their studies on proteins involved in mTOR signaling pathway. Furthermore, Straface et al. [32] hypothesize that differences between sexes may result from differences at the cellular level. Differences between males and females also have been reported by Sharma et al. [33], who demonstrated the lower gene expression of *Pgc1α* in female cerebellar granule neurons (CGNs) than in male mice, resembling our findings in the brain. In contrast, gene expression of *Pgc1α* did not change in the other organs examined in the present study, namely in the kidneys and liver. These findings seem to be quite difficult to explain. However, van Leeuwen et al. [28] reported lack of association between *PGC1α* gene expression and mtDNA content. Moreover, similarly to our current results showing a decrease of *Nrf2* and *Mfn2* mRNA level, gene expressions of related factors – *Nrf1* and *Mfn1* in CGNs – were also decreased in females compared to males, although *Mfn2* mRNA remained unchanged [33]. In Wistar rats, mtDNA content was lower in female brown adipose tissue than in the same tissue of males [34] and had a tendency to be decreased in female liver, although without statistically significant difference [35].

Intriguingly, in other studies, opposite results, i.e., increased level of mitochondrial biogenesis or function in the female sex, was detected. For example, in the Leiden Longevity Study, women had a higher mtDNA content than men [28]. Increased biogenesis of mitochondria may result from female sex hormones administration. Capllonch-Amer et al. [36] reported that 17β-estradiol (E2) up-regulated the markers of mitochondrial biogenesis, dynamics and function in the skeletal muscles in rats. E2 also induced stimulation of mitochondrial biogenesis in white adipocytes *in vitro* [37]. Moreover, there is a wealth of evidence that demonstrates 17β-E2 increases lipid oxidation or improves metabolic parameters (reviewed by [38]). Therefore, one could hypothesize that females may be more efficient in substrate utilization even though they have reduced mitochondrial biogenesis. Females may have more functional reserve so a diminishment of mitochondrial biogenesis or activity may not translate into metabolic perturbations.

One should emphasize that there are clear sex differences in various characteristics of mice with local disruption of the GHR or with global GHR knockout. For example, increased local *Igf1* gene expression in subcutaneous and retroperitoneal white adipose tissue and decreased levels of circulating insulin-like growth factor-binding protein-5 (IGFBP-5) and IGFBP-7 in female LiGHRKO mice compared to controls have been reported [25]. Moreover, List et al. [24] report that alterations in serum leptin and circulating interleukin 6 levels as well as increased total lean body mass are present in female but not in male FaGHRKO mice. In GHRKO dwarfs, significant sex differences in the percent fat mass and in absolute lean mass have been reported [39]. These differences between male and female mice may be related to sex differences in GH secretion pattern and pituitary GH network responses [40]. Plasma GH pattern in males is characterized by high GH pulses occurring with a specific periodicity; in females, GH secretion is less variable with smaller GH pulses and higher interpulse levels [41].

In summary, the role of GH signaling in the regulation of mitochondrial biogenesis appears to be much more complicated than once thought. Local deletion of the GHR in the liver may serve as a very useful experimental animal model for analyzing the role of tissue-specific disruption of GH signaling in the control of processes observed in living organisms. Importantly, sexual dimorphism may play a relevant role in the regulation of mitochondrial biogenesis under conditions of reduced GH signaling. Further studies are required to elucidate the relationships between GH-induced signaling, sex and mitochondria.

## MATERIALS AND METHODS

### Animals

LiGHRKO mice were generated at Ohio University as previously described [25]. Briefly, mice carrying the GHR “floxed” allele were crossed with B6.Cg-Tg(alb-cre)21Mgn/J transgenic mice purchased from Jackson Laboratories (Bar Harbor, ME USA) [24, 25]. Brains, kidneys and livers from approximately 22-month-old male and female wild-type (WT) and liver-specific GHRKO (LiGHRKO) mice were provided from Mayo Clinic, Rochester, MN. The animals comprised four (4) experimental groups: wild-type males (WT-male), liver-specific GHR knockout males (LiGHRKO-male), wild-type females (WT-female) and liver-specific GHR knockout females (LiGHRKO-female), each group consisting of 7 animals.

### RNA extraction and complementary DNA (cDNA) transcription

RNA was extracted from the homogenates of the examined tissues using a miRNeasy Mini Kit (Qiagen, USA) in accordance with the manufacturer's instruction. RNA quantity and quality were analyzed using a NanoDrop 1000 Spectrophotometer (Thermo Scientific, USA). Reverse transcription was performed, and complementary DNA was synthesized using an iScript cDNA Synthesis Kit (Bio-Rad Laboratories, Hercules, CA, USA) according to the manufacturer's instruction.

### Real-time polymerase chain reaction (RT-PCR)

Real-time polymerase chain reaction (RT-PCR) was carried out using the StepOne™ Real-Time PCR System instrument (Life Technologies, USA) with iQ SYBR Green Supermix (Bio-Rad Laboratories, Hercules, CA, USA). The three steps of the PCR included: denaturation at 94°C for 2 minutes, annealing at 62°C for 30 seconds with fluorescence reading, and extension at 72°C for 30 seconds. In addition, a melting curve was done for each reaction to evaluate the potential of nonspecific products. β_2_-microglobulin (B2M), which was previously validated in our laboratory as the most appropriate gene for normalizing the data [12, 13, 42], was used as a housekeeping gene. Gene expression was assessed by measuring steady state levels of mRNA. Relative expression from RT-PCR was calculated using the equation 2^A-B^/2^C-D^ (where A = Cycle Threshold [Ct] number for the gene of interest in the first control sample; B = Ct number for the gene of interest in the analyzed sample; C = Ct number for the housekeeping gene in the first control sample; D = Ct number for housekeeping gene in the analyzed sample). The first control was expressed as 1.00 by this equation, and all other samples were calculated in relation to this value. Then, the results in the control group (WT-males) were averaged. All other outputs were divided by the mean value of the relative expression in the control group to yield the fold change of the expression of genes of interest compared to the control group. For RT-PCR, the primers used are listed in Table 1.

**Table 1:** Primers used for gene expression analyses

Gene	GenBank accession no.	Forward (5′-3′)	Reverse (5′-3′)
*β_2_-microglobulin*	NM_009735	aagtatactcacgccaccca	aagaccagtccttgctgaag
*Pgc1α*	BC066868	tacgcaggtcgaacgaaact	acttgctcttggtggaagca
*Ampk*	AF036535	cacttgtctgcatctctcca	cttgaggaacttgaggatcc
*Sirt1*	AY377984	gtaatgtgaggagtcagcac	ttggacattaccacgtctgc
*Nrf2*	U20532	tcagtgactcggaaatggag	ttcacgcataggagcactgt
*Mfn2*	NM_133201	ccacaaagtgagtgaacgtc	atccaccagaaagctggtgc
*CoxIV*	NM_009941	acagcccttggcttgatgta	tggcctgaaagcttccacta

### Statistical analysis

The data are expressed as mean ± SEM. To evaluate the main effects of the genotype and sex, we used two-way analysis of variance (ANOVA). For analyzing differences between group means, we used a Bonferroni post-hoc test. A value of p<0.05 was considered significant. All statistical calculations were conducted using SPSS version 17.0 (SPSS, Chicago, IL) with α=0.05. All graphs were created using Prism 4.02 (GraphPad Software, San Diego, CA).

To statistically analyze differences between males and females (a potential significant gender effect) we pooled all males (WT-male and LiGHRKO-male mice) and all females (WT-female and LiGHRKO-female mice) (see the Results section). Such statistical analysis may allow detect some potentially significant differences, which couldn't been found when analysis between particular experimental groups was only performed. We would like also to emphasize that the employed method (pooling samples from male and female mice) is fully justified and statistically correct because the groups that were being combined (pooled) [i.e., WT-males and LiGHRKO-males (pooled into male group) vs. WT-females and LiGHRKO-females (pooled into female group)] were not significantly different from each other.
